# Editorial: Women in science: ophthalmology 2023

**DOI:** 10.3389/fmed.2024.1454872

**Published:** 2024-07-29

**Authors:** Menaka C. Thounaojam, Gemma Caterina Maria Rossi

**Affiliations:** ^1^Department of Cellular Biology and Anatomy, James and Jean Culver Vision Discovery Institute, Medical College of Georgia at Augusta University, Augusta, GA, United States; ^2^Department of Ophthalmology, James and Jean Culver Vision Discovery Institute, Medical College of Georgia at Augusta University, Augusta, GA, United States; ^3^James and Jean Culver Vision Discovery Institute, Medical College of Georgia at Augusta University, Augusta, GA, United States; ^4^La Struttura Complessa Oculistica, Fondazione IRCCS Policlinico San Matteo, Pavia, Italy; ^5^Ambulatorio Glaucoma, ASST Bergamo Est, Ospedale Locatelli, Bergamo, Italy

**Keywords:** ophthalmology, women in science, gender disparities in STEM, vision research, academic publishing

In the ever-evolving landscape of science and medicine, the pivotal role of women remains indispensable yet often undervalued. Despite constituting an equal share of STEM undergraduates, UNESCO Institute for Statistics (UIS) data from 2016 underscores that women account for < 30% of science, technology, engineering, and mathematics researchers (1). This underrepresentation persists within the realm of medicine, including the domain of Ophthalmology, with women significantly absent from upper levels of leadership. However, the narrative is changing, propelled by the remarkable contributions of pioneering women reshaping vision research and Ophthalmology (2, 3).

Even as strides are made, women in science continue to face multifaceted challenges, from income disparities to systemic biases. Initiatives aiming to amplify the visibility of women in STEM fields have emerged, including the inception of prestigious awards. Nevertheless, the COVID-19 pandemic has laid bare the persisting fault lines, exacerbating existing gender disparities and impeding the progress of women in academic pursuits (4, 5). The “leaky pipeline” metaphor vividly captures the myriad barriers obstructing the advancement of women in STEM, underscored by gender bias and structural obstacles (6).

The pandemic has cast a stark light on the gender gap in academic publishing, with women's contributions to Ophthalmology and vision research suffering a setback. Despite decades of gradual ascent, recent findings reveal a difficulty in the upward trajectory of female participation, particularly in senior roles. This Research Topic is a testament to our commitment to rectifying this imbalance and celebrating the achievements of women in Medicine, specifically within Ophthalmology. Across 11 meticulously curated articles, authored or co-authored by women from diverse corners, we spotlight the groundbreaking research shaping the future of eye care and vision science.

This Research Topic consists of seven articles published following a peer review of many submissions. In a thorough cross-sectional study involving 193 glaucoma patients, researchers delved into the intricate relationship between the visual field index (VFI) and the quality of life (QoL), considering various clinical and demographic factors. They discovered significant correlations between VFI and QoL measures, even after adjusting for confounding variables such as age, gender, comorbidities, treatment, and visual acuity. Notably, the study highlighted that higher VFI scores were associated with improved QoL outcomes, suggesting that VFI could serve as a valuable tool in assessing and monitoring the wellbeing of glaucoma patients, thus emphasizing its relevance in clinical practice for evaluating treatment efficacy and patient outcomes (Rossi et al.).

Another notable investigation focused on patient satisfaction and outcomes related to serum eye drops (SED) for dry eye syndrome. Through a comparative study between autologous SED (Auto-SED) and patient-tailored SED (PT-SED), researchers uncovered that while both types of SED improved dry eye symptoms for most patients, those receiving PT-SED showed decreases in some quality-of-life measures. Additionally, the study revealed positive feedback regarding SED and vial packaging, with areas for improvement identified, underscoring the importance of patient input in optimizing treatment strategies (Gemelli et al.).

Moreover, a university-based study explored the relationship between optic disc morphology, axial length, and retinal vessel distribution in healthy young adults. Using spectral-domain optical coherence tomography angiography, researchers identified associations between optic disc rotation, tilt, vessel density, and ocular parameters. These findings shed light on the significance of considering optic disc morphology when assessing retinal vessel density, particularly in individuals with myopia, offering insights that could enhance diagnostic accuracy and treatment planning in ophthalmology practice (Chen et al.).

Furthermore, research aimed to establish a core outcome set (COS) for postoperative outcomes following cataract surgery with monofocal intraocular lenses (IOLs). This initiative, driven by healthcare professionals and patients, aimed to specify clinical outcomes and patient-centered aspects essential for evaluating different technologies in monofocal IOLs. By incorporating diverse perspectives, this approach seeks to optimize healthcare resources by considering both clinical effectiveness and patient preferences, thus offering a comprehensive tool for assessing treatment outcomes (Tarricone et al.).

In a separate investigation, the accuracy of patients' perceived causes of primary open-angle glaucoma (POAG) was evaluated, alongside its implications for illness perceptions, medication adherence, and quality of life (QoL). The study revealed that while a significant proportion of patients reported accurate causes, there were no significant differences in medication adherence and QoL between accuracy groups. Understanding patients' perceptions of disease causes remains crucial for effective disease management, highlighting the need for patient-centered care approaches in glaucoma management (Choe et al.).

Lastly, discussions centered on genetic and genomic studies in glaucoma, emphasizing their pivotal role in identifying new genetic loci associated with the disease and understanding its genetic susceptibility across diverse populations. Despite promising advancements in gene-based screening for personalized treatment plans, challenges persist in achieving high diagnostic yields, particularly for adult-onset glaucoma. Nevertheless, ongoing research endeavors aim to leverage genetic insights to develop personalized risk assessments and innovative therapeutic approaches, offering hope for improved outcomes in glaucoma management (Tirendi et al.).

Similarly, a comprehensive review explored the intricate relationship between gut microbiota-derived metabolites and ocular health, challenging conventional beliefs regarding organ sterility and highlighting the dynamic nature of the gut-eye axis. By examining the role of specific metabolites such as short-chain fatty acids (SCFAs) and bile acids (BAs), the review underscored their significant contributions to ocular pathologies, thus advocating for a targeted approach in manipulating microbiome-related metabolites for optimal ocular health outcomes. This nuanced strategy aims to bypass the limitations associated with broad interventions, offering a more efficient pathway toward desired therapeutic outcomes in optimizing gut and ocular health (Nguyen et al.).

In conclusion, the ongoing efforts to highlight and support the contributions of women in ophthalmology and vision research are vital in addressing the gender disparities that persist in STEM fields. This Research Topic underscores the importance of their groundbreaking work, offering a comprehensive overview of the diverse and innovative research led by women. By showcasing these achievements, we aim to inspire further progress and ensure that the momentum gained in advancing gender equity in science and medicine continues to flourish. Through continued advocacy and support, we can pave the way for a more inclusive and equitable future in ophthalmology and beyond.

## Author contributions

MT: Writing – original draft, Writing – review & editing. GR: Writing – original draft, Writing – review & editing.
